# The Interplay Between Tissue Niche and Macrophage Cellular Metabolism in Obesity

**DOI:** 10.3389/fimmu.2019.03133

**Published:** 2020-01-22

**Authors:** Sabine Daemen, Joel D. Schilling

**Affiliations:** ^1^Department of Medicine, Washington University School of Medicine, St. Louis, MO, United States; ^2^Department of Pathology and Immunology, Washington University School of Medicine, St. Louis, MO, United States

**Keywords:** obesity, insulin resistance, non-alcoholic fatty liver disease, macrophages, metabolism, liver, adipose tissue

## Abstract

Obesity is associated with the development of metabolic diseases such as type 2 diabetes and non-alcoholic fatty liver disease. The presence of chronic, low-grade inflammation appears to be an important mechanistic link between excess nutrients and clinical disease. The onset of these metabolic disorders coincides with changes in the number and phenotype of macrophages in peripheral organs, particularly in the liver and adipose tissue. Macrophage accumulation in these tissues has been implicated in tissue inflammation and fibrosis, contributing to metabolic disease progression. Recently, the concept has emerged that changes in macrophage metabolism affects their functional phenotype, possibly triggered by distinct environmental metabolic cues. This may be of particular importance in the setting of obesity, where both liver and adipose tissue are faced with a high metabolic burden. In the first part of this review we will discuss current knowledge regarding macrophage dynamics in both adipose tissue and liver in obesity. Then in the second part, we will highlight data linking macrophage metabolism to functional phenotype with an emphasis on macrophage activation in metabolic disease. The importance of understanding how tissue niche influences macrophage function in obesity will be highlighted. In addition, we will identify important knowledge gaps and outstanding questions that are relevant for future research in this area and will facilitate the identification of novel targets for therapeutic intervention in associated metabolic diseases.

## Introduction

Adipose tissue and liver contain tissue resident macrophages that are indispensable for tissue homeostasis. Tissue macrophages can either be derived from primitive hematopoiesis in the embryonic yolk-sac or from definitive hematopoiesis from the fetal liver or bone marrow-derived monocytes (1). Fate-mapping studies have revealed that the majority of tissue-resident macrophages are initially derived from the embryonic yolk-sac and maintain via self-renewal; however, this varies amongst tissues (1). Although circulating monocytes contribute to the resident macrophage pool in some tissues, monocyte-derived macrophages (MdMs) predominantly enter tissues in states of tissue injury or inflammation. Cellular origin may be one factor that contributes to the functionality of tissue macrophages; however, these cells display a high degree of plasticity and can readily adapt their functional state in response to environmental cues. Indeed, the transcriptome of resident macrophages from different tissues is very distinct (2), despite similar cellular origins. Thus, tissue niche is arguably the most important driver of resident macrophage function. However, the cellular and molecular pathways that program the resident macrophage identity are just beginning to be understood (3, 4). Although tissue resident macrophages share common functions, including tissue remodeling and clearance of cellular debris, they can also exert specific tissue function. For example, alveolar macrophages regulate pulmonary surfactant homeostasis (5) and liver Kupffer cells (KCs) play an important role in iron metabolism (6). However, it is largely unclear if and how changes in the tissue microenvironment with obesity affect tissue macrophage function and what the consequences are for tissue homeostasis and pathology.

Traditionally, macrophage activation has been described by the two-dimensional M1 and M2 spectrum, i.e., classical or alternative activation, respectively. Recently, this paradigm has been replaced by the concept that there is a continuous spectrum of macrophage activation that can be shaped by tissue environment, cell programming, and activating stimuli. In large part, this notion has arisen from data using fate mapping and phenotyping techniques combined with systems biology (7). Therefore, identifying macrophage subsets in health and disease requires a combined approach based on surface marker expression, (single cell) RNA sequencing and functional/metabolic phenotyping. In the setting of obesity there are extensive shifts in macrophage number and activation state in the adipose tissue and liver and these changes contribute to metabolic disease progression. Macrophage lipotoxicity has been well-described and contributes to complications of obesity such as atherosclerosis, insulin resistance, and non-alcoholic fatty liver disease (NAFLD). Metabolic programming of macrophages themselves can also influence cell function and is particularly relevant to inflammation associated with nutrient excess. However, the interplay between macrophage cellular metabolism and tissue niche during the development of obesity and its complications is poorly understood. This review will address the current knowledge on altered macrophage dynamics in adipose tissue and liver in obesity. Furthermore, we will elaborate on the interplay between systemic metabolic perturbations and cellular metabolism in macrophages in the setting of excess lipids. The potential of targeting macrophage metabolic function to modulate obesity complications will also be discussed.

## Macrophage Dynamics in Obese Adipose Tissue

### Accumulation of Adipose Tissue Macrophages in Obesity

**Figure 1:**
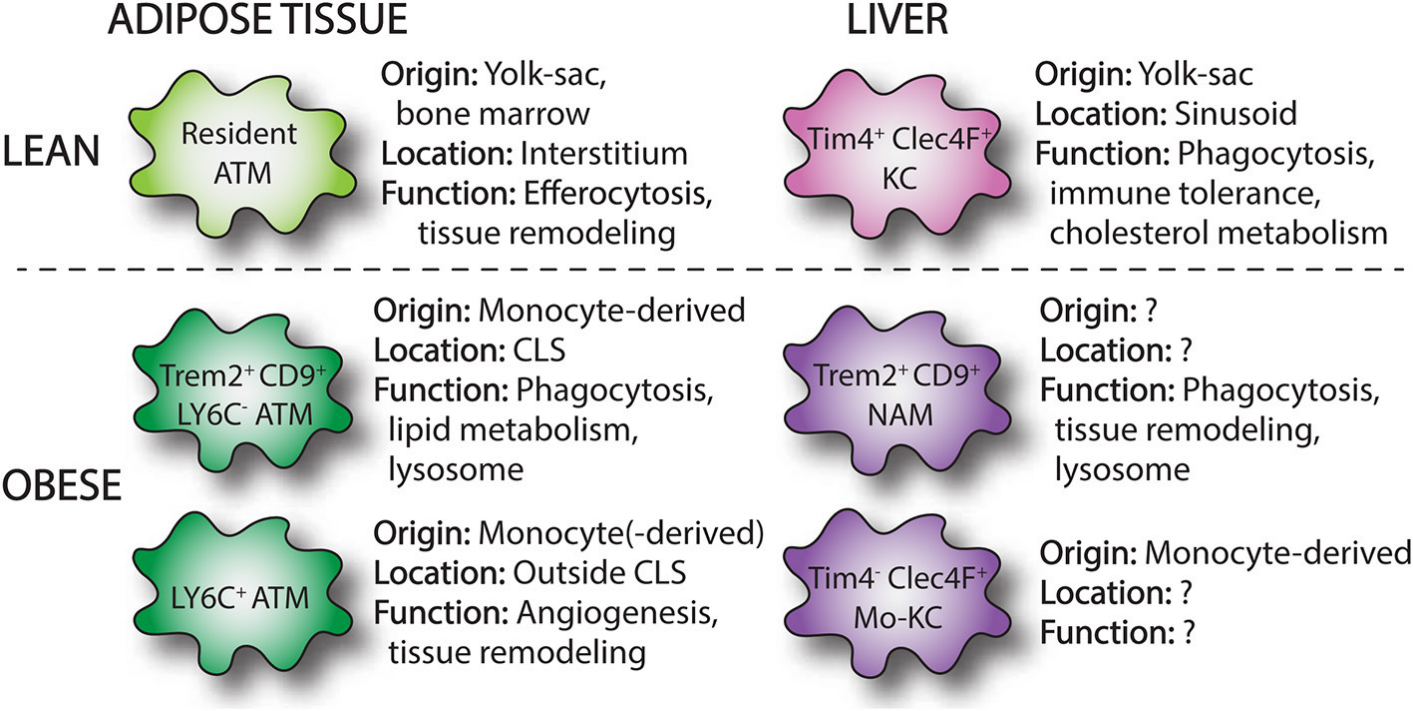
Overview of macrophages subsets in lean and obese adipose tissue and liver. Single-cell RNA sequencing has identified macrophage subpopulations that accumulate in liver and adipose tissue in the setting of obesity. These obese adipose tissue macrophages (ATMs) and NASH-associated macrophages (NAMs) are derived from infiltrating monocytes and display distinct surface markers (Trem2, CD9) and functions compared to resident tissue macrophages. In the liver, loss of resident KCs in NASH induces the appearance of monocyte-derived KCs (mo-KCs), which likely fill the empty KC niche and may exert similar functions to resident KCs.

Lean adipose tissue contains resident macrophages that reside between adipocytes and alongside vascular structures. These resident adipose tissue macrophages (ATMs) have various roles in adipose tissue homeostasis including efferocytosis (i.e., removal of dead adipocytes), lipid buffering, and adipogenesis (Figure 1) (8). In addition, resident ATMs are important for the expansion and contraction of adipose tissue that occurs with changes in fat mass (9). Although the origin and maintenance of resident ATMs is less well-understood it was recently shown that resident ATMs in healthy murine adipose tissue are predominantly derived from embryonic yolk-sac precursors and can self-renew via proliferation (10, 11). However, bone marrow-derived macrophages also contribute to the resident ATM pool (10, 12). Excess ATMs can be eliminated by type 1 innate lymphoid cells (13), thereby maintaining a homeostatic ATM number and potentially preventing unwanted inflammation.

In obesity, adipose tissue expands and there is a marked accumulation of macrophages (14, 15). While in lean adipose tissue ATMs represent about 5–10% of stromal cells, in obese adipose tissue this increases up to 40–50% in mice (14). In humans, a similar increase in adipose macrophage content has been demonstrated (16–18). The expansion of macrophages with obesity was initially thought to result from the recruitment of Ly6C^hi^ monocytes to adipose tissue (14). In line with this, higher levels of monocyte chemoattractants are present in obese adipose tissue, most notably monocyte chemoattractant protein-1 (MCP-1, also known as CCL2) (19). In addition, labeling studies have shown that circulating monocytes can enter obese adipose tissue via mechanism that is largely dependent on CCR2, the receptor for CCL2 (20). However, subsequent data has also shown that in addition to recruitment, local proliferation of ATMs also contributes to the increase in macrophage numbers observed in obese adipose tissue (21, 22). Importantly, these models are not mutually exclusive and likely both contribute as recruited monocyte-derived cells can also proliferate. Other local mechanisms may also contribute to increased macrophage numbers, including increased tissue retention. This is possibly mediated by Netrin-1 (23), which has been implicated in the regulation of cellular migration of various cell types.

In addition to increases in number, the localization and organization of ATMs within the tissue changes with obesity. Whereas, in lean adipose tissue ATMs are interstitially spaced, in the obese setting ATMs form clusters. These sites of macrophage accumulation are known as crown-like structures (CLS), in which macrophages appear to surround apoptotic adipocytes (24, 25). Interestingly, local proliferation of ATMs occurs predominantly in CLS (21). The role of these macrophages will be discussed more in the next section.

### ATM Phenotype in Obesity

Macrophage phenotype also changes in obese adipose tissue. Macrophages in murine lean adipose tissue have been characterized as F4/80^+^, CD11b^+^, CD206^+^, CD301^+^ cells. In obese adipose tissue, CCR2^+^/Ly6C^hi^ monocytes enter the tissue where they can differentiate to ATMs and/or proliferate (26). Early studies demonstrated that increased expression of CD11c is a common feature of ATMs present in obese adipose tissue, particularly in monocyte-derived cells (27, 28). Although CD11c can be help identify recruited ATMs, this receptor is also expressed by adipose tissue dendritic cells (DCs). Thus, additional markers, such as the macrophage marker CD64, are necessary to distinguish CD11c^+^ ATMs from DCs.

As mentioned above, the activation of state of macrophages in obesity has often been described based upon the two-dimensional M1 and M2 activation states (29). In this respect, a switch from an anti-inflammatory M2 phenotype to proinflammatory M1 phenotype was thought to occur in obese adipose tissue (30). In support of this model, lean adipose tissue macrophages have an “M2-like” phenotype characterized by gene expression of anti-inflammatory genes, such as Ym1, arginase 1, and IL-10 (30, 31), whereas F4/80^+^, CD11c^+^ ATMs express genes ascribed to classically activated M1 macrophages, including iNOS and TNFα (31). However, this concept was initially challenged by data obtained from flow-sorted macrophages revealing that upregulation of lysosomal genes was the strongest transcriptional signal in ATMs from obese mice. Thus providing evidence that tissue remodeling and phagocytosis may be more relevant functions of ATMs than the release of pro-inflammatory cytokines (32). This concept has subsequently been confirmed by two recent studies from independent labs that used single cell RNA sequencing (scRNA-seq) to demonstrate substantial differences between murine and human ATMs in obesity compared to M1 or M2 phenotypes (33, 34). In one of these studies, the tetraspanin CD9 together with Ly6C were identified as markers to define three distinct subsets of ATMs; Ly6C^+^, Ly6C^−^CD9^−^, and Ly6C^−^CD9^+^ (33). The Ly6C^+^ population of cells likely represents monocytes, whereas the Ly6C^−^ populations are true ATMs. Interestingly, CD11c expression was variable within the three subpopulations but was highest in the CD9^+^ macrophages. Importantly, these three ATM/monocyte subsets displayed distinct tissue localization and gene expression patterns. Ly6C^+^ monocytes had a uniform tissue distribution outside of CLS and were adipogenic, whereas CD9^+^ ATMs specifically resided within CLS, had high amounts of intracellular lipid and expressed proinflammatory genes (Figure 1). When CD9^+^ ATMs were transplanted into lean mice they induced expression of inflammatory genes in the adipose tissue. Interestingly, lipid-laden CD9^+^ ATMs were also detected in human obese adipose tissue and localized to CLS (33). Subsequently, Jaitin et al. also used a scRNA-seq approach and confirmed that CD9^+^ ATMs accumulate in obese adipose tissue and localize to adipose tissue CLS (34). In this study, these macrophages were referred to as Lipid-Associated Macrophages (LAMs) due to their location around lipid droplets and their high expression of genes involved in phagocytosis and lipid metabolism (Figure 1). In addition, LAMs express high levels of the lipid receptor Trem2 and knock out of this receptor in bone marrow cells worsened the metabolic consequences of obesity. This observation suggests that although these ATMs have some pro-inflammatory characteristics they actually play a beneficial role in obese adipose tissue. This duality of macrophage function in obesity is also supported by data from mice lacking the enzyme NADPH oxidase 2 (NOX2) in macrophages. In these mice, loss of NOX2 diminishes inflammatory cytokine production, but also impairs the clearance of dead adipocytes upon high-fat diet (HFD) feeding. As a result, myeloid-NOX2 KO mice have decreased adipose tissue inflammation and improved glucose tolerance early in disease, but develop profound insulin resistance and steatohepatitis upon prolonged HFD. Thus, although ATMs in obesity have been viewed as pathologic, mainly due to their pro-inflammatory potential, they also perform beneficial functions such as the clearance of dead adipocytes and buffering of excess fatty acids (35). This concept is important to consider when designing and interpreting experimental results as the timepoint of analysis can yield divergent results. Together this data supports a model whereby CD9^+^/Trem2^+^ ATMs can play either pro-inflammatory or reparative roles in adipose tissue based on disease severity.

Human ATMs are characteristically CD14^+^/CD16^−^ and express markers CD68, CD163, CD204, and CD206 in lean adipose tissue. It has been recognized that in humans, ATMs barely express markers used for M1 and M2 classification, such as iNOS and arginase-1 (36). Rather human ATMs in obese adipose tissue express a mixed phenotype illustrated by expression of both CD11c, an M1 marker, and CD206 and CD163, markers of M2 activation (37–39). CD11c^+^, CD206^+^ macrophages have been shown to correlate with insulin resistance (38), whereas the number of CD11c^+^, CD163^+^ cells associated with BMI (39). Furthermore, CD163 was found to be the only marker to track with HOMA-IR (40). Analogous to the murine system, human ATMs appear to simultaneously have the potential to release pro-inflammatory cytokines including TNFα, IL-6, IL-1, and MCP-1 while also expressing factors associated with tissue remodeling and homeostasis such as IL-10 and TGFβ (37, 41). As mentioned above, CD9^+^ ATMs resembling LAMs have been described in humans; however, further work will be necessary to assess whether these immune cells play similar functions in both species.

In summary, obesity drives ATM accumulation and shifts in gene expression that are not well-captured by the classical M1/M2 model. More in depth analysis with detailed phenotyping of obese ATMs in both mice and humans has revealed an alternative model of ATM activation in adipose tissue characterized by an upregulation of cell adhesion, lipid metabolism, and lysosomal genes. The cues that lead to ATM activation appear to be related to metabolic factors such as free fatty acids, lipoproteins, glucose and insulin; a state referred to as metabolically activated macrophages (32, 35, 42). Further understanding of the metabolic signaling events that shape adipose tissue macrophage phenotype including their adaptive and detrimental roles will be critical to unlock the therapeutic potential of immune modulation during obesity.

## Macrophage Dynamics in Liver

### Resident and Infiltrating Macrophages

In contrast to the adipose tissue, the liver contains a robust population of resident macrophages known as Kupffer cells (KCs). KCs represent 35% of all liver non-parenchymal cells and are the largest tissue population of resident macrophages in the body. They are largely derived from erythromyeloid progenitors of the embryonic yolk sac and maintained by self-renewal (43, 44). These resident macrophages appear critical for liver (immune) homeostasis (Figure 1). KCs are intravascular and line the endothelium of the liver sinusoids where they represent a first line of defense to gut-derived pathogens, microbes, and toxins. Together with other immune cells, KCs are important for mediating immune tolerance, at least in part by the expansion of regulatory T-cells (45). In addition, their long cytoplasmic extensions can extend into the extravascular space and allow for contact with liver parenchymal cells. Beyond immune homeostasis, KCs can regulate the metabolism of iron, bilirubin, as well as cholesterol (46). In mice, KCs are traditionally characterized as F4/80^hi^, CD11b^int^, CD68^+^ cells. Recently, additional surface markers have been described that specifically identify KCs from other myeloid cells in the liver including T-cell immunoglobulin, mucin domain containing 4 (Tim4), and C-type lectin domain family 4 member F (Clec4F) (Figure 1) (47, 48). In addition, KCs lack expression of CCR2 and Cx3-chemokine receptor 1 (Cx3cr1) (12). MdMs are derived from bone-marrow hematopoietic stem cells and are present in small numbers in the liver under physiologic conditions. Murine monocytes infiltrating the liver are characterized as CD11b^+^, Cx3cr1^+^, Ly6c^+^. In addition, CCR2 is expressed at high level and regulates monocyte influx into the liver. Upon entry into the liver, monocytes downregulate Ly6C and undergo a maturation process to MdMs, which is poorly understood but appears to be dependent upon the local tissue environment. Monocytes become a significant contributor to the liver macrophage pool in circumstances of KC depletion or tissue injury/inflammation (48). Upon experimental depletion of KCs, it has been demonstrated that monocytes can give rise to bona fide KCs which repopulate the empty KC niche (47, 48). Although these monocyte-derived KCs resemble their yolk sac brethren, there are some transcriptional programs that remain distinct (42). However, it is yet clear to what extent this differentiation process occurs with liver pathology such as NAFLD.

Human KCs as well as MdMs are less well-characterized and distinctive markers are lacking. KCs have often been characterized by their expression of CD68 and CD14. However, MdMs also express these markers. A recent study using RNA sequencing revealed two distinct populations of CD68^+^ liver macrophages in the healthy human liver, with one population having an inflammatory gene expression pattern and the second population was characterized by an immunotolerance phenotype (49). These populations could be distinguished by unique expression of MARCO in the immunotolerance population. More recently scRNA-seq data from humans with cirrhosis suggests that KCs also express CD163 and Tim4 in addition to MARCO (50), a phenotype that is analogous to KCs in mice (47). However, the ontogeny of these liver macrophages and whether these macrophage subtypes include MdMs in addition to true KCs is not known. The markers for monocytes and MdMs in the human liver are less well-understood, but include CCR2, Cx3cr1, SA100A2, and CD14 (50). Ongoing investigation will continue to establish the relationship between murine liver macrophage populations and their human counterparts.

### Macrophages in Obesity and NAFLD

Obesity manifests in the liver as non-alcoholic fatty liver disease (NAFLD). Although it was initially tempting to speculate that similar inflammatory responses might occur in both adipose tissue and liver with obesity, it is now apparent that these organs respond in very different ways to lipid stress. NAFLD is characterized by excessive hepatic lipid accumulation, which can result in hepatocyte injury and cell death. This can trigger an inflammatory response and progression to non-alcoholic steatohepatitis (NASH), advanced fibrosis, cirrhosis, and hepatocellular carcinoma (51). Macrophages are important mediators of the inflammatory response which underlies the progression of NAFLD to NASH. Dietary models of NAFLD in mice have demonstrated an increase in liver macrophages number during NAFLD development (52, 53), but this finding has not been consistent with many studies also reporting no increase in total macrophage count (54, 55). Regardless, an important role of liver macrophages in NASH development has been demonstrated by depletion of liver macrophages by clodronate or gadolinium chloride, which have been shown to attenuate hepatic steatosis and decrease inflammation (53, 56–58).

Numerous studies have suggested macrophages take on a proinflammatory phenotype in NAFLD, which is likely mediated by a variety of environmental signals. To this end it has been shown that excessive accumulation of toxic lipids by KCs in the steatotic liver led to dysregulated lipid handling and increased pro-inflammatory gene expression (59). Inflammatory activation of KCs can also be regulated by danger signals released from hepatocyte damage and death (46) and by increased bacterial products from the gut due to gut dysbiosis in obesity (60). In addition to resident KCs, CCR2^+^, Cx3cr1^+^, Ly6C^hi^ monocytes infiltrate the liver in experimental models of NASH and appear to give rise to “pro-inflammatory” macrophages (61). However, in many of these earlier studies KCs could not be distinguished from MdMs and therefore the specific roles of resident vs. recruited cells in liver inflammation is not well-defined.

CCR2 is a critical chemokine receptor for monocyte recruitment into tissue. While genetic or pharmacologic disruption of CCR2 appears to attenuate liver inflammation with obesity this has not been consistent across CCR2 loss of function models (62–64). As such, the role of resident vs. recruited cells in NASH progression has remained controversial. In addition to CCR2, the chemokine receptor CXCR3 has also been described to play a role in monocyte recruitment (65). CCL2 can be secreted by KCs, hepatocytes, stellate cells and sinusoidal endothelial cells to facilitate monocyte recruitment. KC depletion by clodronate suppresses the infiltration of monocytes into the liver, potentially due to decreased CCL2 release (53, 66); however, these studies are challenging to interpret as clodrolip also depletes monocytes directly. Liver sinusoidal endothelial cells also upregulate VCAM-1 in response to inflammation thereby facilitating adhesion of monocytes (67). Related to this, it has been reported that under lipotoxic stress hepatocytes release extracellular vesicles which are enriched with the integrin ITGβ_1_, a VCAM-1 ligand, which subsequently enhances monocyte adhesion to sinusoidal endothelial cells (68).

Monocyte infiltration is also an important feature of human NASH and is especially seen in patients with more severe disease states and advanced liver fibrosis (69, 70). Similar to what has been described in the adipose tissue, a key feature of both human and mice NASH is the presence of macrophage aggregates termed crown-like structures (64, 71). These macrophage aggregates appear to surround dead or dying hepatocytes with large lipid droplets and colocalize with areas of fibrosis and activated stellate cells (64). The number of CLS positively correlates with the extent of fibrosis, suggesting that these structures may serve as hot spots for stellate cell activation (71). Thus, it is likely that the interplay between liver macrophages and hepatic stellate cells activation contributes to the tissue fibrosis observed in NASH (72–74). However, it remains unclear what drives the formation of hepatic CLS, what cells contribute to these aggregates, and what functions these cells have in the progression of NASH and fibrosis development.

On the other hand, macrophages with an anti-inflammatory and restorative phenotype have been demonstrated to reduce NASH severity (75–77). KCs themselves can promote anti-inflammatory responses including the secretion of IL-10, which can trigger apoptosis of inflammatory macrophages (75). Furthermore, infiltrating macrophages can adopt a restorative phenotype characterized by high Cx3cr1 expression and expression of genes involved in matrix remodeling (74). In human NASH increased expression of markers associated with alternative activation of macrophages has also been detected (78). Thus, similarly as described for adipose tissue, it is likely that liver macrophages perform beneficial roles in an attempt to restore liver injury.

Although it is clear that both resident and monocyte-derived macrophages play an important role in the progression of simple steatosis to NASH, the dynamics and function of these distinct macrophage subsets in the pathogenesis of NASH is unclear. The heterogeneity amongst MdMs themselves also appears to be more complex than appreciated. In part this may be related to the use of suboptimal macrophage markers and the assumption that all resident macrophages are F480^hi^, CD11b^int^ while MdMs are CD11b^hi^, F4/80^int^. In support of this concept, it was recently reported that resident KCs are depleted upon MCD-diet feeding even though F4/80^hi^ cells remain constant due to influx of MdMs. Moreover, this study also revealed that one potential fate of monocyte-derived cells is to become monocyte-derived KCs (mo-KCs, Figure 1) (79). More recently, scRNA-seq analysis in HFD fed mice (amylin diet) uncovered a unique subset of Trem2^+^, CD9^+^, Gpnmb^+^ macrophages in the liver referred to as NASH-associated macrophages (NAMs, Figure 1). Gene expression analysis points toward a potential role of NAMs in the clearance of apoptotic and lipid debris as well as tissue remodeling (80). Interestingly, NAMs display some overlapping surface markers and transcriptional programs with LAMs. Whether these cells function in a similar way as LAMs in tissue remodeling is not known.

Future studies will be required to dissect the dynamic shifts that occur in macrophage subsets during NASH pathogenesis. In addition, the specific cues that induce macrophage polarization in NAFLD are not completely understood and the interplay between metabolic cues and macrophage function may be crucial for NASH progression.

### The Role of Macrophages in the Interaction Between Liver and Adipose Tissue

Chronic oversupply of nutrients to the adipose tissue can eventually lead to adipocyte dysfunction and an inability to store these excess nutrients. This can evoke increased ectopic lipid deposition in other tissues including liver, skeletal muscle, and heart (81). ATMs can directly increase adipose tissue lipolysis through the release of specific cytokines, including TNFα, IFN-γ, and IL-1β (82, 83) and thereby enhance the lipid flux to the liver. Further evidence for adipose-liver interactions comes from a recent study demonstrating that acute adipocyte death induces rapid CCR2^+^ macrophage infiltration into the adipose tissue. These macrophages enhance lipolysis via modulation of epinephrine and norepinephrine levels. Interestingly, this wave of adipocyte death also causes acute secondary liver injury which is dependent upon CCR2^+^ macrophage infiltration into the liver (84). Although fatty acids serve as one important mediator that links adipose tissue and liver, the crosstalk between these tissues goes beyond lipids. In addition, obese adipose tissue secretes a range of other nutrients, adipokines, and inflammatory molecules which can promote pathogenic events in the liver (85). This is exemplified by leptin, an adipokine that signals via hepatic macrophages to induce hepatic stellate cell activation and liver fibrosis (86).

Inflamed adipose tissue has also been associated with the development of NASH. Moreover, there is increasing evidence that adipose inflammation is directly linked to liver pathology. For example, surgical removal of epididymal adipose tissue in HFD-induced obesity was shown to reduce circulating inflammatory mediators and attenuate NASH progression (87). In addition, ATM expansion and expression of inflammatory mediators precedes hepatic inflammation in HFD-induced obesity, suggesting that macrophage responses in the adipose tissue may be upstream of NASH development. In line with this concept, data from a recent study demonstrated that transplanting adipose tissue from obese mice increased liver macrophage content and NASH severity compared to adipose tissue from lean mice. This response was attenuated when ATMs were depleted from the adipose tissue prior to transplantation (88). Although most studies have focused on how adipose inflammation can modulate the pathogenesis of NASH, the interaction between liver and adipose inflammation is likely reciprocal. In fact, it has been demonstrated that preventive depletion of liver macrophages inhibits adipose tissue inflammation upon HFD feeding (89). The interplay between adipose and hepatic inflammation is likely also relevant in human NASH. This is supported by data showing that the expression of several inflammatory mediators, including TNFα, IL-8, and CCL-3, as well as ATM number in the adipose tissue is associated with NAFLD and NASH severity and liver fibrosis in patients (90–92).

Future studies investigating the crosstalk between the macrophages in liver and adipose tissue during obesity are anticipated to reveal additional mediators linking adipose tissue inflammation and NASH. In particular, the role of macrophage-secreted exosomes and their cargo are now recognized as important mediators of crosstalk between macrophages in distant tissues as well as between macrophages and parenchymal cells. In this respect, ATMs from obese murine adipose tissue have been shown to secrete exosomes containing miRNA-29a and miRNA-155 (93, 94). These secreted exosomes are taken up by adipocytes but also by other tissues, including liver and skeletal muscle. This was shown to result in impaired glucose tolerance and decreased insulin sensitivity. Thus, exosome secretion may be an important paracrine and autocrine signal by which tissue macrophages can influence local and systemic metabolism. In addition to macrophage-derived exosomes, these vesicles are also secreted by adipocytes and hepatocytes. Importantly, adipocyte-derived exosomes have been shown to affect macrophage activation and polarization, with predominantly an increase in inflammatory cytokine production (95, 96). In addition, when exosomes isolated from VAT of obese patients were applied to hepatocyte or hepatic stellate cell lines *in vitro*, uptake of these vesicles induced an upregulation of the TGFβ signaling pathway and expression of genes related to extracellular matrix deposition (97). This indicates a possible direct role of adipose tissue-derived exosomes in NASH progression, although future studies will be needed to determine the extent to which this occurs *in vivo*. As studies have shown that obesity is associated with increased circulating extracellular vesicles, this may be of particular importance in obesity and the development of its metabolic complications (98). Dissecting the molecules and pathways involved in inter-organ crosstalk in the metabolic syndrome has the potential to drive the discovery of new therapeutic targets.

## Metabolic Reprogramming of Macrophage Function

### Glucose Metabolism

Increased glucose uptake and upregulation of glycolysis, the so-called glycolytic switch, is associated with pro-inflammatory macrophage activation and is important for classical inflammatory macrophage effector functions including phagocytosis, pro-inflammatory cytokine production and reactive oxygen species (ROS) generation (99, 100). The rapid increase in glucose uptake in activated macrophages is facilitated by upregulation of glucose transporter 1 (GLUT1). In some instances, GLUT1 overexpression has been shown to increase glucose metabolism and augment the secretion of inflammatory mediators and ROS production (100). However, these findings have not been recapitulated in primary macrophages where GLUT1 overexpression does not augment inflammatory cytokine release, despite increasing glucose uptake and utilization (101). Thus, glucose metabolism and macrophage inflammation are related but dissociable responses, suggesting the interplay between glycolysis/glycolytic metabolites and inflammatory signaling pathways ultimately shapes cell function. In the context of metabolic disease, GLUT1 expression is upregulated in both adipose tissue and liver of HFD-fed rats and colocalizes specifically with macrophages present in CLS in both organs (100). Whether perturbations of GLUT1 impact ATM-mediated functions and/or insulin resistance is not known.

The NLRP3 inflammasome has recently been recognized as an important driver of inflammation in many complications of obesity including atherosclerosis, insulin resistance, and NASH (102, 103). In macrophages, NLRP3 complex assembly and IL-1β transcription are both sensitive to perturbations in glucose metabolism. As an example, hexokinase-1-dependent glycolysis promotes NLRP3 inflammasome activation and IL-1β secretion, a response mediated by mTORC1 (104). In addition, the transcription factor Hypoxia-inducing factor 1-α (HIF1α) appears to be another important link between glucose metabolism and NLRP3 inflammasome regulation. In activated macrophages HIF1α is stabilized by the tricarboxylic acid cycle (TCA) cycle intermediate succinate, which accumulates in part due to increased glycolytic flux (105, 106). Subsequently, HIF-1α induces the expression of GLUT1 and other glycolytic genes (107) and may directly upregulate gene expression of IL-1β via PKM2 (105, 108, 109). The relevance of this pathway in metabolic disease is supported by data demonstrating that mice lacking HIF1α are protected against HFD-induced adipose tissue inflammation (110). Moreover, there is evidence that HIF-1α is upregulated in macrophages in both mice and humans with NASH (111). In addition to the inflammasome, glycolytic flux can also influence the production of other pro-inflammatory cytokines, such as TNFα. This can occur via the bi-functional enzyme glyceraldehyde 3-phosphate dehydrogenase (GAPDH), which negatively regulates TNFα expression by inhibiting its translation. As such, when the rate of glycolysis increases GAPDH is recruited for its metabolic function and the break on TNFα translation is released (112). Together, these data demonstrate a key role for glycolytic enzymes and intermediates in the regulation of macrophage-mediated inflammatory responses.

Upregulation of glucose uptake also feeds substrate into the pentose phosphate pathway (PPP) which is also upregulated in inflammatory macrophages (113). The PPP is important for the generation of amino acids and ribose for protein and nucleotide synthesis as well as NADPH, which is essential for NADPH oxidase-mediated ROS production, fatty acid synthesis, and generation of glutathione. The regulation of the PPP is mediated by carbohydrate kinase-like protein (CARKL) in primary murine and human macrophages. CARKL is suppressed in response to LPS activation, resulting in increased flux through the PPP (114). To date the inflammatory and metabolic effects of inhibiting flux through the PPP in ATMs or liver macrophages is less well-understood.

Although increased glycolytic flux is a hallmark of inflammatory macrophage activation, glycolysis is also required for alternatively macrophage activation. Mechanistically, it has been shown that IL-4 and M-CSF activate mTORC2, which subsequently upregulates glycolysis via IRF4 (115). However, unlike inflammatory macrophages where the products of glycolysis are frequently channeled away from the mitochondria, in alternative activation glycolysis supports the TCA cycle and mitochondrial oxidative phosphorylation (OXPHOS). In line with this notion, the inhibition of glycolysis with 2-deoxyglucose (2-DG) reduced OXPHOS and attenuated early M2 marker expression in response to IL-4 (116). However, there is also evidence that 2-DG can decrease OXPHOS directly which may also explain some of these findings (117).

In summary, although glucose metabolism is associated with classical inflammatory macrophage activation, it is also required for alternative macrophage activation. Thus, selective modulation of glucose metabolism is a more viable approach for disrupting macrophage inflammation. Further investigation will also be necessary to unravel the complex interplay between macrophage glucose handling and the hyperglycemia that frequently exists in states of obesity and insulin resistance.

### Lipid Metabolism

In contrast to the stimulation of glycolytic metabolism that occurs in inflammatory macrophages, increased lipid uptake and oxidation is a hallmark of alternative macrophage activation induced by IL-4. In this system, IL-4 increases mitochondrial fatty acid oxidation (FAO) and this metabolic response appears to be necessary for macrophages to acquire an M2 phenotype (118). This is supported by data demonstrating that inhibiting FAO or OXPHOS with compounds like etomoxir, oligomycin, or the mitochondrial uncoupler FCCP, attenuates the expression of classic M2 polarization markers (e.g., arginase, YM1, Rentla) (118, 119). Mechanistically, the metabolic reprogramming induced by IL-4 is dependent upon the transcriptional effects of STAT6 and PGC-1β (119). The nuclear receptor transcription factors PPARγ and PPARβ/δ have also been reported to contribute to the upregulation of genes involved in lipid uptake, FAO, and mitochondrial OXPHOS. In general, the source of fatty acids to fuel this response derives from exogenous triacylglycerol substrate, which is taken up via scavenger receptor CD36 and broken down by lysosomal acid lipase (LAL)-mediated lipolysis (118). In line with this, attenuation of lipolysis by orlistat decreases oxidative respiration and reduces alternative macrophage polarization. Moreover, orlistat or knockout of LAL has been shown to decrease oxidative respiration and reduce alternative macrophage polarization. Although exogenous fatty acids appear to be the predominant source of lipid, triglyceride derived from fatty acid synthase can also be used to fuel OXPHOS in some circumstances. Despite strong evidence that IL-4 mediated alternative activation of macrophages requires FAO, whether these lipid metabolic pathways are as important for reparative macrophage phenotypes *in vivo* is not known.

Other lipid metabolic regulators that have been shown to influence macrophage inflammatory function include fatty acid transporter protein FATP1 and FABP4/5 (120, 121). Deletion of FATP1 in cultured macrophages induces a metabolic switch toward glycolysis and primes macrophages for proinflammatory functions. *In vivo*, loss of FATP1 in bone marrow derived cells was associated with increased HFD-induced weight gain, glucose intolerance, and adipose tissue inflammation. In contrast, deletion or inhibition of FABP4 or FABP5 attenuates lipid-induced inflammation and improves metabolic phenotypes *in vivo* (121). Further mechanistic links between FAO and macrophage function came from experiments in which macrophages were engineered to overexpress carnitine palmitoyl transferase (CPT)-1. In this system, CPT1 overexpression increased FAO, decreased triglyceride content and dampened pro-inflammatory cytokine production (122). Together these data indicate that modulating lipid handling has potential as a strategy to shift macrophage function in obesity related diseases.

The regulation of cellular lipid metabolism is also controlled by several key nuclear receptor transcription factors and their co-activators. Peroxisome proliferator-activated receptors (PPARs) act as lipid sensors and modulate both lipid storage and utilization (123). Both PPARβ/δ and PPARγ have been shown to influence macrophage polarization, as ablation of either inhibits IL-4 stimulated alternative macrophage activation (76, 124, 125). With HFD feeding, myeloid-specific PPARγ knock-out mice have increased weight gain, adiposity, and glucose intolerance, which was paralleled by decreased expression of classic alternative macrophage activation markers and increased adipose tissue inflammation (124). Myeloid-specific deletion of PPARβ/δ also resulted in increased weight gain, adiposity, and insulin resistance upon HFD feeding. In both liver and adipose tissue, proinflammatory gene expression was increased and M2 marker expression reduced (125). In addition, steatosis in the liver was more severe in the knockout animals. Together this data supports the idea that targeting lipid metabolic pathways by enhancing PPAR activity in macrophages could be utilized as a strategy to modulate obesity complications. It is also worth noting that disrupting PPAR activity in macrophages may also accelerate the development of obesity itself.

Macrophages also express other transcription factors that regulate cholesterol and lipid metabolism including liver X receptors (LXR), CCAAT enhancer binding proteins (C/EBPs), and sterol regulatory element binding proteins (SREBPs). Of interest, KCs express high levels of LXRα, which acts as cholesterol sensor and regulates intracellular cholesterol levels by expression of cholesterol efflux transporters ABCA1 and ABCG1 (126, 127) as well as apolipoproteins as ApoE and ApoC (128, 129). Interestingly, LXR is necessary for the maintenance of KCs in the liver (130). *In vitro* studies have shown that ligands of LXR receptors inhibit expression of inflammatory mediators as iNOS, COX-2, MMP-9, and IL-6 (131) and can upregulate expression of arginase II which potentiates anti-inflammatory effects (132). SREBPs are considered master regulators of cholesterol and lipid synthesis. In general, they coordinate the balance between lipid uptake and *de novo* lipogenesis/cholesterol biosynthesis. Besides their role in lipogenesis, SREBP1a enhances expression of nlrp1a, a key component of inflammasome activation and IL-1β release in macrophages *in vitro* (133). Deletion of SREBP-1a in cultured macrophages results in increased inflammatory gene expression following TLR4 stimulation, likely due to a lack of anti-inflammatory unsaturated fatty acid production (134). These findings argue that LXRs and SREBPs link lipid metabolism to macrophage inflammatory function; however, the role of these pathways in obesity-related inflammation is less clear.

C/EBPα and C/EBPβ are also widely expressed in macrophages and have been implicated in regulating glucose and lipid metabolism (135). C/EBPβ appears to promote alternative macrophage activation in skeletal muscle macrophages (136). Germane to this finding, myeloid-specific knock-out of C/EBPα led to increased adiposity in chow-fed mice, indicating an important role of macrophage C/EBPα in energy homeostasis under physiological conditions (137). Upon HFD, C/EBPα deletion in mice protected against diet-induced insulin resistance mainly due to preserved skeletal muscle insulin sensitivity. Macrophage polarization markers revealed both M1 and M2 markers to be downregulated (137). Similarly, HFD-fed mice transplanted with bone-marrow lacking C/EBPβ had reduced expression of inflammatory markers and decreased macrophage content in adipose tissue. This inflammatory phenotype was associated with improved insulin sensitivity. Of relevance, C/EBPβ can induce the expression of PPARγ and LXRα in primary macrophages; however, it is unclear if this is related to the detrimental metabolic effects observed in C/EBPβ KO mice (138).

Recently, the requirement of FAO for alternative macrophage activation has been questioned. Primary macrophages lacking CPT2 have reduced rates of FAO, but have no defect in IL-4-induced alternative activation (139). In addition, blocking FAO oxidation by use of etomoxir does not affect IL-4-induced differentiation of primary human macrophages. In fact, in some circumstances FAO can potentiate pro-inflammatory responses including inflammasome activation. In this respect, FAO via CPT1 can promote activation of the NLRP3 inflammasome via effects on by NADPH oxidase 4 (NOX4) (140). This can be stimulated by fatty acids as palmitate and may require mitochondrial ROS production (141, 142). In line with this data, deletion of PPARγ in macrophages leads to a reduction IL-1β production and release in response to NLRP3 activators, an effect mediated by IFN-β (143). Thus, FAO and resultant metabolic byproducts play a complex role in the regulation of macrophage reparative and inflammatory functions. Together these data demonstrate a multifaceted relationship between lipid metabolism and inflammation in metabolic disease. Regardless, these proof-of-concept studies provide evidence that lipid metabolic pathways may be viable targets for modulating macrophage function.

### TCA Cycle

In addition to enhanced glycolysis, a disrupted TCA cycle is an important feature of classically activated macrophages (113). The “broken” TCA cycle results in decreased production of α-ketoglutarate and an accumulation of citrate and succinate. Citrate accumulates as a result of reduced isocitrate dehydrogenase (Idh1) expression in inflammatory macrophages. Toll-like receptor stimulation also induces the expression of the mitochondrial citrate carrier (CIC) in macrophages. CIC regulates citrate efflux from mitochondria to cytosol where it can be used for *de novo* lipogenesis, production of proinflammatory lipid mediators (prostaglandin E2) and synthesis of NO (144). Citrate is also converted to acetyl-CoA by ATP-dependent citrate lyase (ACLY) and this can enhance pro-inflammatory gene expression via histone acetylation (145).

Pyruvate dehydrogenase kinase (PDK) has been postulated to be important for M1 polarization as it reduces entry of carbons from glycolysis into the TCA cycle by inhibiting pyruvate dehydrogenase (PHD). Indeed, pharmacological inhibition of PDK2 and−4 blocks inflammatory activation of cultured macrophages (146). Myeloid-specific ablation of PDK2/4 using a bone-marrow transplant approach led to reduced weight gain, improved glucose tolerance and reduced adiposity and liver fat when fed a HFD. In addition, HFD-induced ATM accumulation as well as adipose tissue inflammatory expression was diminished. On the contrary, recent data has also shown that glucose-derived pyruvate conversion to mitochondrial acetyl-CoA by pyruvate dehydrogenase (PHD) is maintained during LPS stimulation and is important for pro-inflammatory macrophage activation as it fuels citrate production (147).

Citrate can also serve as a precursor for itaconate, which is produced by the enzyme immunoresponsive gene 1 (Irg1). This metabolite subsequently inhibits succinate dehydrogenase (SDH) and elevates succinate levels (148, 149). Succinate stabilizes HIF-1α, which as mentioned above upregulates IL-1β production (105). Moreover, SDH is also a subunit of complex II of the electron transport chain, where oxidation of succinate is coupled to reduction of ubiquinone. Increased oxidation of succinate by SDH results in production of mitochondrial ROS, also supporting HIF-1α stabilization and inflammatory gene expression (150). In more recent data it has been shown that succinate is also a ligand for the succinate receptor SUCNR1. This is relevant as activated macrophages can secrete succinate, which influences macrophage polarization in both an autocrine and paracrine manner via SUCNR1 (151, 152). The importance of this signaling pathway was demonstrated by creating mice with a myeloid-specific knock-out of SUCNR1. These animals were more susceptible to diet-induced obesity and had worsened glucose tolerance (152). This was paralleled by an increased inflammatory molecular signature in ATMs of these mice, suggesting that extracellular succinate might play a role in dampening inflammatory responses. Analysis of WAT explants from lean and obese subjects also showed this succinate-SUCNR1 pathway was disturbed in obese human WAT. In summary, TCA cycle metabolites are important determinants of metabolic programming through their allosteric properties and their ability to directly activate signaling pathways. This “metabokine” function of TCA intermediates has been proposed to be an important regulatory mechanism of inflammatory responses (153). Harnessing the signaling properties of TCA cycle intermediates holds promise as a tool for modulating macrophage metabolism in disease.

### Amino Acid Metabolism

Glutamine is one the most important amino acids to fuel macrophage activation. Interestingly, glutamine has different metabolic fates in M1 and M2-like macrophages. In inflammatory macrophages, glutamine-derived glutamate fuels in to the TCA cycle for succinate production via GABA production (105). Hence it contributes to the effects of succinate accumulation on pro-inflammatory macrophage polarization. Furthermore, glutamine has been shown to enhance macrophage lipotoxicity, as removal of glutamine from the nutrient microenvironment attenuates macrophage cell death and inflammasome activation upon exposure to excess saturated fatty acids (154). M2/IL-4 stimulated macrophages also have increased glutamine levels, which occurs predominantly via the activity of glutamine synthetase (GS) (155). Glutamine also contributes to M2 polarization via glutamine-derived α-ketoglutarate, which is essential for FAO as well as epigenetic programming during alternative macrophage activation (156). Thus far, most studies on the effects of glutamine on the polarization of macrophages have been limited to cultured murine macrophages. Although patients with obesity and diabetes have been shown to have reduced serum glutamine levels, it remains to be further studied if and to what extent glutamine metabolism affects tissue macrophage polarization in both mice and humans (157).

The amino acid L-arginine also has divergent roles in macrophage polarization. L-arginine is an essential precursor for the production of NO via iNOS, which is induced during inflammatory macrophage activation. On the other hand, metabolism of L-arginine by arginase is associated with alternative macrophage activation and appears important for tissue repair (158). It is likely that other amino acids can modulate macrophage biology and this is an area that will require further investigation.

### Macrophage Regulation of Obesity and Insulin Resistance

**Figure 2:**
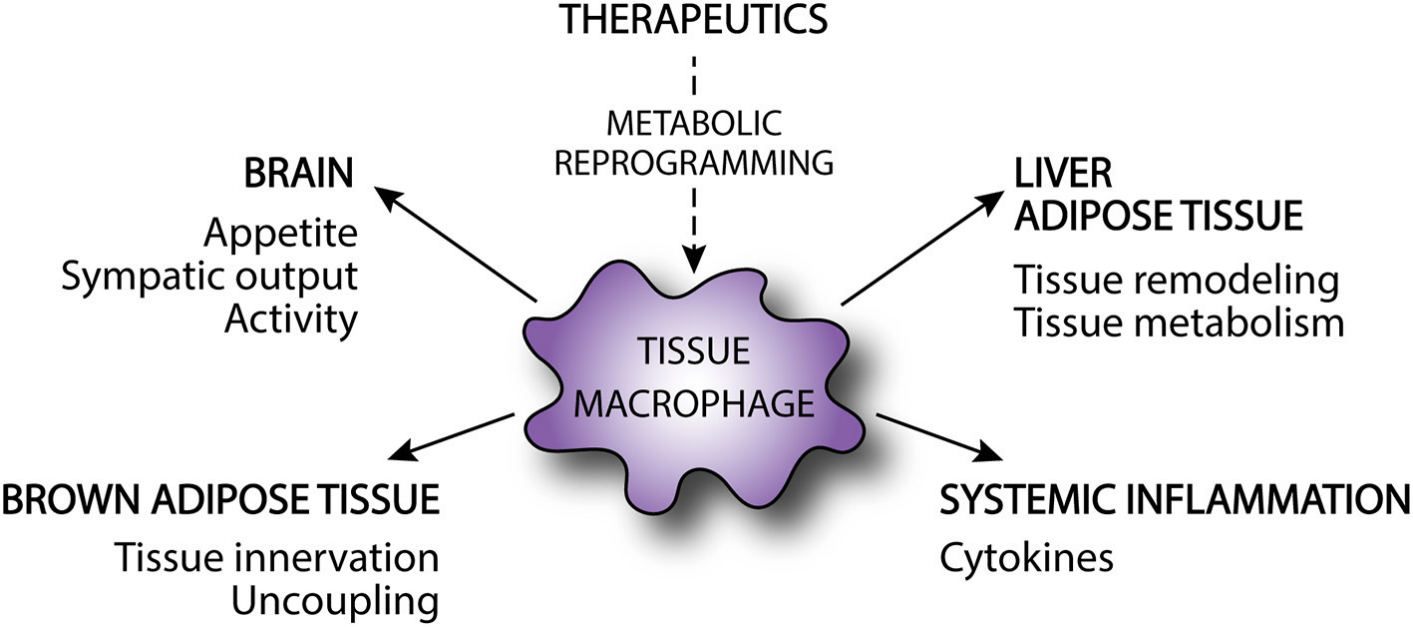
Modulation of obesity and metabolic disorders by tissue macrophages. Tissue macrophages can affect obesity and the development of associated metabolic disorders by affecting energy intake and energy expenditure via brain, brown adipose tissue, and skeletal muscle. Alternatively, tissue macrophages modulate development of metabolic disorders in obesity by regulating adipose tissue and liver metabolism and tissue remodeling as well as by contributing the systemic inflammation. Metabolic reprogramming via targeted therapeutics may alter macrophage activation and subsequently improve metabolic disease.

As reviewed above, targeting important metabolic pathways in macrophages has the potential to alter not only macrophage function, but also systemic metabolism. However, the exact mechanisms by which tissue macrophages regulate whole-body metabolism are largely unknown and this remains an important knowledge gap in the field. As discussed above, it is clear that macrophages can interact with parenchymal cells in the adipose and liver in ways that modulate systemic glucose homeostasis (Figure 2) (159). In addition, the release of pro-inflammatory cytokines from metabolically activated macrophages can also promote skeletal muscle and hepatic insulin resistance. However, there is also evidence that whole-body metabolism can be modulated via inflammatory signaling that occurs locally in the brain (Figure 2). As an example, it was recently shown that HFD causes accumulation of pro-inflammatory microglia in the brain of mice. Moreover, pharmacologic or genetic disruption of NF-κB-dependent microglial activation decreased hyperphagia and reduced diet-induced obesity (160). The exact pathways through which microglia influence energy balance are unclear, but could be related to changes in appetite, activity level, or sympathetic nervous output. Of importance, sympathetic output regulates energy expenditure in part by modulating brown adipose tissue (BAT) activity. Tissue resident macrophages in BAT also contribute to the metabolism of catecholamines, which influences thermogenesis and energy homeostasis (Figure 2) (161).

## Conclusions and Future Perspectives

**Figure 3:**
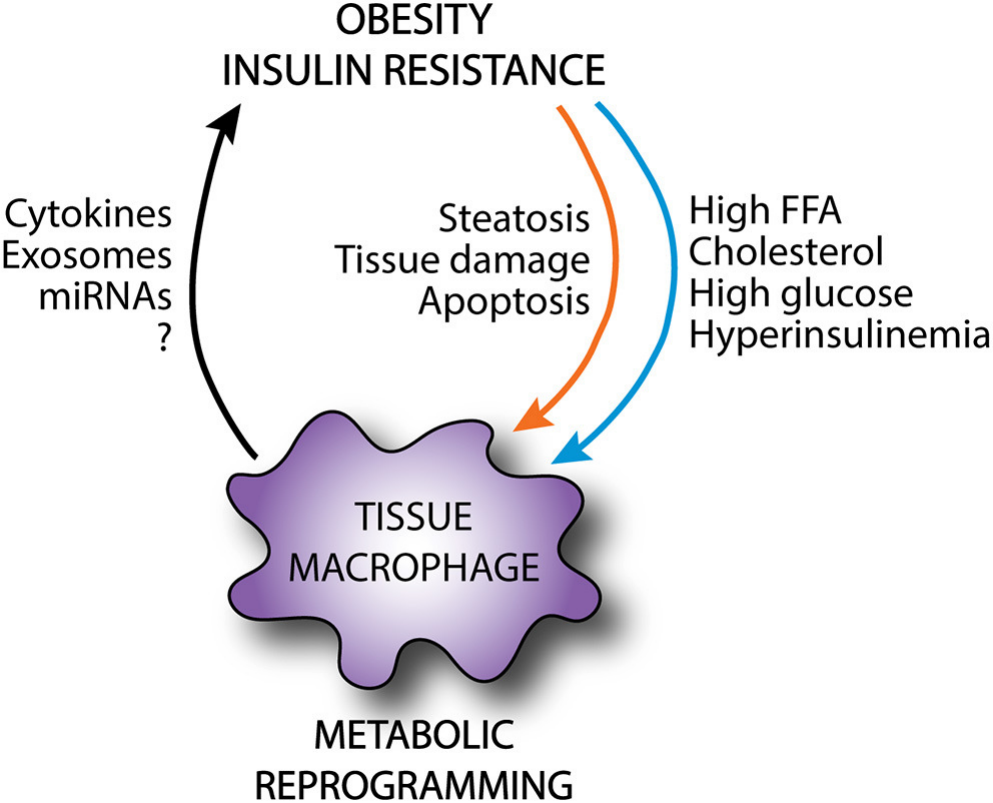
Metabolic reprogramming of tissue macrophages in obesity. In obesity, tissue macrophages are exposed to an excessive and altered nutrient environment and receive a variety of danger signals from damaged parenchymal cells. This can rewire the metabolic programming of tissue macrophages, altering the activation state of these cells. Although these macrophages may exert protective functions as shielding and clearance of apoptotic cells, they can also release local and systemic mediators that can further exacerbate metabolic disease.

Obesity exposes tissue macrophages to a microenvironment that consists of excess nutrients as well as stress signals from damaged and apoptotic parenchymal cells. These environmental cues can activate macrophages and rewire their cellular metabolism in ways that may promote maladaptive inflammation and macrophage dysfunction. Macrophage activation may initially be beneficial to facilitate the removal of apoptotic cells, clear excess lipids, and restore tissue homeostasis. However, aberrant metabolic reprogramming of macrophages in the setting of sustained lipid-induced tissue injury likely provokes pathologic macrophage phenotypes. When this occurs the macrophage secretome may act locally and/or systemically to drive metabolic disease development (Figure 3). This concept is particularly true in tissues that are faced with a high metabolic burden in obesity, such as adipose tissue and liver. In order to harness the therapeutic potential of re-programming macrophage metabolism for human disease, it will be essential to dissect the mechanistic pathways by which macrophages improve or worsen obesity-related complications.

Although it is established that perturbing macrophage cellular metabolism can improve metabolic disease phenotypes, the precise cellular and molecular details remain poorly understood. In part this is due to the fact that much of the data in this field comes from knockout or overexpression of key metabolic genes in the context of *in vitro* models or non-selective myeloid knock-out systems. Ultimately, elucidating the heterogeneity of macrophage populations in different organs and their relation to metabolic disease will allow more specific therapeutic targeting. However, our understanding of this has been limited by the lack of tissue-specific macrophage markers to identify and manipulate resident macrophages in different tissues. As such, most studies have used generic myeloid knock-out models, such as LysM-Cre or bone-marrow transplantation. The identification of unique markers of tissue macrophages through the use of sophisticated unbiased approaches such as scRNA-seq, has the potential to open up new avenues to investigate the role of distinct macrophage subsets in tissue and whole-body metabolism. As an example, it has recently been shown that liver resident KCs uniquely express the surface receptors Tim4 and Clec4f. This discovery facilitated the development of Clec4f-Cre transgenic mice, which will enable the study KC specific functions in liver pathology and metabolic disease (130).

Another challenge to advancing this field relates to the impact of variations in genetic background, microbiota composition, and research environment on phenotypes in metabolic disease research (162, 163). As a consequence, it is commonplace that genetic and pharmacologic disruption of inflammatory/metabolic pathways in macrophages produce contradictory results. In order to minimize the influence of these variables it is critical to utilize littermate controls and to co-house wild type and knockout mice in the same cage. The same principle holds true for pharmacologic intervention where mice from both treatment groups should be mixed together in cages. These interventions will reduce the influence of gene-environment interactions that confound data interpretation. In addition, the use of human systems to validate mouse model observations will be vital to improve the identification of relevant therapeutic targets for modulating macrophage metabolism.

The pathogenesis of obesity-related metabolic disorders, such as insulin resistance is highly complex and involves metabolic perturbations in multiple organs. Tissue macrophages secrete a plethora of factors that may affect macrophage polarization and subsequently tissue function in distant organs. Evidence already exists to support the concept that adipose tissue macrophages can direct relevant pathological events in the liver in the setting of obesity. Conversely, it is attractive to hypothesize that signaling from liver macrophages to adipose tissue also occurs. It will be important to identify the key molecules involved in inter-organ crosstalk and to evaluate their effects on tissue macrophage biology.

The objective of this review was to discuss our current understanding of macrophage diversity in metabolically relevant tissues and to consider the intersection of these principles with the regulation of macrophage cellular metabolism. Although significant progress has been in these areas, further investigation and integration of these fields will be necessary to harness the therapeutic potential of re-programming macrophage metabolism in obesity and diabetes. In addition, defining the molecular basis of crosstalk between macrophages and parenchymal cells in adipose tissue, liver, and the brain will also require more study. Another important issue to resolve in this area is whether targeting macrophage metabolism can attenuate pathologic inflammation during obesity without disabling the fundamental roles of these immune cells in tissue repair and the maintenance of homeostasis. Eventually, the use of targeted small molecules or nanoparticles are attractive modalities to modulate macrophage function. Although there is much yet to be learned, metabolic targeting of macrophage biology holds promise as therapeutic strategy to modulate metabolic disease.

## Author Contributions

SD and JS conceptualized and wrote the manuscript.

### Conflict of Interest

The authors declare that the research was conducted in the absence of any commercial or financial relationships that could be construed as a potential conflict of interest.

## Abbreviations

ATM: adipose tissue macrophage
CLS: crown-like structures
DCs: dendritic cells
FAO: fatty acid oxidation
HFD: high fat diet
KCs: Kupffer cells
LAMs: Lipid-associated macrophages
MdMs: monocyte-derived macrophages
NAFLD: non-alcoholic fatty liver disease
NAMs: NASH-associated macrophages
NASH: non-alcoholic steatohepatitis
OXPHOS: oxidative phosphorylation
PPP: pentose phosphate pathway
scRNA-seq: single cell RNA sequencing
TCA cycle: tricarboxylic acid cycle.
